# Steroid-triggered, cell-autonomous death of a *Drosophila *motoneuron during metamorphosis

**DOI:** 10.1186/1749-8104-6-15

**Published:** 2011-04-27

**Authors:** Ari Winbush, Janis C Weeks

**Affiliations:** 1 Department of Biology, Institute of Neuroscience, University of Oregon Eugene, OR, 97403-1254, USA; 2 Section of Molecular and Computational Biology, Department of Biological Sciences, University of Southern California, Los Angeles, CA 90089, USA

## Abstract

**Background:**

The metamorphosis of *Drosophila melanogaster *is accompanied by elimination of obsolete neurons via programmed cell death (PCD). Metamorphosis is regulated by ecdysteroids, including 20-hydroxyecdysone (20E), but the roles and modes of action of hormones in regulating neuronal PCD are incompletely understood.

**Results:**

We used targeted expression of GFP to track the fate of a larval motoneuron, RP2, in ventral ganglia. RP2s in abdominal neuromeres two through seven (A2 to A7) exhibited fragmented DNA by 15 hours after puparium formation (h-APF) and were missing by 20 h-APF. RP2 death began shortly after the 'prepupal pulse' of ecdysteroids, during which time RP2s expressed ecdysteroid receptors (EcRs). Genetic manipulations showed that RP2 death required the function of EcR-B isoforms, the death-activating gene, reaper (but not hid), and the apoptosome component, Dark. PCD was blocked by expression of the caspase inhibitor p35 but unaffected by manipulating Diap1. In contrast, aCC motoneurons in neuromeres A2 to A7, and RP2s in neuromere A1, expressed EcRs during the prepupal pulse but survived into the pupal stage under all conditions tested. To test the hypothesis that ecdysteroids trigger RP2's death directly, we placed abdominal GFP-expressing neurons in cell culture immediately prior to the prepupal pulse, with or without 20E. 20E induced significant PCD in putative RP2s, but not in control neurons, as assessed by morphological criteria and propidium iodide staining.

**Conclusions:**

These findings suggest that the rise of ecdysteroids during the prepupal pulse acts directly, via EcR-B isoforms, to activate PCD in RP2 motoneurons in abdominal neuromeres A2 to A7, while sparing RP2s in A1. Genetic manipulations suggest that RP2's death requires Reaper function, apoptosome assembly and Diap1-independent caspase activation. RP2s offer a valuable 'single cell' approach to the molecular understanding of neuronal death during insect metamorphosis and, potentially, of neurodegeneration in other contexts.

## Background

Metamorphosis of the fruit fly, *Drosophila melanogaster*, entails the transformation of a crawling, feeding larva into an adult capable of flight and reproduction. During this process the larval nervous system is reorganized to accommodate new adult-specific behaviors through neuronal remodeling, the development of previously quiescent adult-specific imaginal neurons, and the elimination of obsolete larval neurons by programmed cell death (PCD) [1,2]. Neuronal death likewise accompanies embryogenesis, but the extent to which underlying causes and mechanisms of neuronal PCD are shared during these two periods, or with PCD in other cell types, is largely unexplored [3,4].

**Figure 1:**
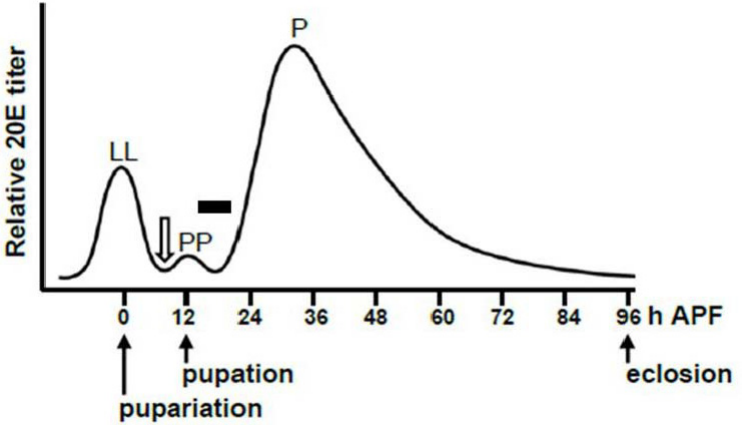
**Endocrine events during *Drosophila *metamorphosis**. Timeline illustrates changes in ecdysteroid levels during the larval-pupal-adult transformation at 25°C. The late larval (LL) pulse of 20-hydroxyecdysone (20E) triggers pupariation, with subsequent development timed by hours after puparium formation (h-APF). The prepupal (PP) pulse at approximately 12 h-APF triggers pupation; the pupal (P) pulse drives development of the adult fly. Emergence of the adult (eclosion) occurs at approximately 96 h-APF. Open arrow indicates when neuronal cultures were prepared (8 h-APF; see Results). Filled bar shows period when abdominal RP2s undergo PCD (approximately 14 to 20 h-APF; see Results). Adapted from [12] based on hormone titers from [7,72].

Metamorphosis is regulated in large part by steroid hormones termed ecdysteroids, including the active metabolite, 20-hydroxyecdysone (20E). 20E exerts its actions via three isoforms of the ecdysteroid receptor (EcR): EcR-A, EcR-B1 and EcR-B2 [5]. The specific sequence of hormonal changes that drive metamorphosis in *Drosophila *are shown in Figure 1. A 'late larval pulse' transforms the wandering larva into a white prepupa at puparium formation. A smaller 'prepupal pulse' approximately 10 hours after puparium formation (h-APF) initiates pupation, whose hallmark, head eversion, occurs at approximately 12 h-APF. A prolonged 'pupal pulse' beginning at approximately 24 h-APF drives the remaining development of the adult fly. As adult eclosion (emergence) approaches, ecdysteroids decline and remain low or absent during early adult life [6,7].

A role for ecdysteroids in postembryonic neuronal remodeling and PCD has been demonstrated in *Drosophila *and in another well-studied insect species, the hawkmoth *Manduca sexta *[1]. In *Drosophila*, the remodeling of larval motoneurons, mushroom body neurons and FMRFamide-expressing thoracic ventral neurons is regulated by the late larval pulse and pupal pulse of ecdysteroids [8-16]. The PCD of a subset of larval ventral neurons that express the neuropeptide Corazonin (vCrz) appears to be triggered by the late larval pulse, via EcR-B isoforms [17]. In *Manduca*, the remodeling or death of larval abdominal motoneurons is regulated by the prepupal and pupal pulses of ecdysteroids [18-23]. The decline in ecdysteroids at the end of the pupal stage in both *Drosophila *and *Manduca *triggers PCD of neurons not needed during the adult stage[2,24,25]. *Drosophila *neurons in this category include 'type II' neurons identifiable by their strong EcR-A expression, and a subset of neurons that express the neuropeptide crustacean cardioactive peptide [26,27]. The death of type II and crustacean cardioactive peptide neurons requires both an ecdysteroid decline and an unidentified descending signal [25-28]. *Manduca *likewise has neurons that require these two cues to initiate PCD after eclosion [29,30].

In *Drosophila*, PCD has been shown to be controlled through a balance between death-activating and death-inhibiting proteins. The core cell death machinery includes effector caspases which, following their activation by initiator caspases, degrade cellular proteins [31]. In *Drosophila*, the initiator caspase Dronc (*Drosophila *Nedd2-like caspase) likely governs most PCD [32-36]. Dronc autoprocessing and activation requires recruitment into an apoptosome formed by the *Drosophila *Apaf-1-related killer (Dark) protein [37-40]. Animals carrying *dark *mutations show PCD defects similar to those seen in *dronc *mutants [41-43]. Caspases are normally inhibited by the *Drosophila *inhibitor of apoptosis protein (Diap1) through ubiquitination [44-48]. The canonical cell death pathway involves the inactivation and degradation of Diap1 by proteins encoded by the genes *reaper, hid*, and *grim *[49-51]. The inactivation of Diap1 by Reaper, Hid or Grim allows Dronc to accumulate and be recruited into apoptosomes [38,44,52,53].

In *Manduca*, the most detailed information regarding ecdysteroid-induced neuronal PCD has come from the segment-specific death of larval accessory planta retractor (APR) motoneurons during metamorphosis [20]. The death of a segmental subset of APRs at pupation is triggered directly and cell-autonomously by the rise in ecdysteroids during the prepupal peak, as demonstrated by placing the motoneurons in cell culture and exposing them to appropriate changes in 20E levels [54-56]. Likewise, PCD of the remaining APRs, which occurs following eclosion, is a direct, cell-autonomous response to the decline of ecdysteroids at the end of the pupal stage [24].

Delineation of molecular pathways by which ecdysteroids activate postembryonic PCD ideally requires an experimental system in which neurons of interest are direct targets of ecdysteroids, genes/proteins involved in PCD are known, and the EcRs and death genes can be genetically manipulated. The APRs of *Manduca *fulfill the first criterion but largely lack the other features. In contrast, *Drosophila *offers the latter two features but lacks identified neurons whose deaths unambiguously result from direct ecdysteroid action. The present experiments made use of RP2 motoneurons, which have been studied extensively in other contexts [57-59]. We found that a segmental subset of RP2s, located in abdominal neuromeres A2 to A7, undergoes PCD at the onset of metamorphosis in direct response to the rise in ecdysteroids during the prepupal pulse, and that EcRs and previously identified death genes participate in RP2's demise. This experimental system provides an opportunity to investigate the chain of events linking steroid hormone action to the PCD of an individually identified neuron during postembryonic development.

## Results

### GFP labeling to track neurons during metamorphosis

**Figure 2:**
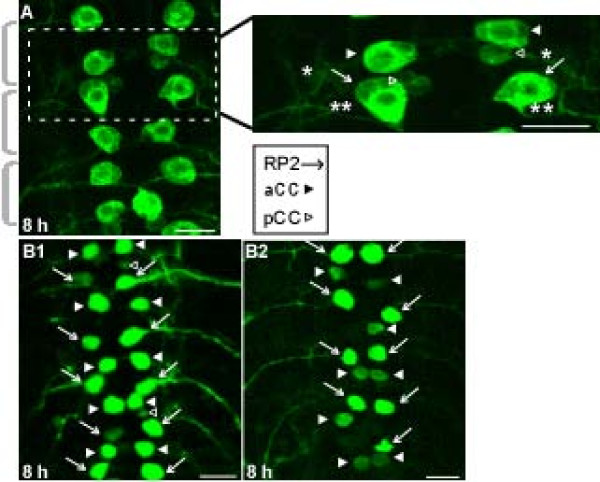
**GFP expression to identify and track neurons**. **(A) **Left: confocal image showing a repeating pattern of GFP-labeled neurons along the anterior-posterior axis of the abdominal region of a ventral ganglion at 8 h-APF. Grey brackets denote neuromeres. For this and all subsequent confocal images, abdominal neuromeres are shown with anterior up, h denotes h-APF, and RP2s, aCCs and pCCs are marked by arrows, filled arrowheads, and open arrowheads, respectively (see key below inset). The dashed box outlines the enlarged inset at right, illustrating GFP labeling in bilateral pairs of aCCs, RP2s and more faintly labeled pCCs. aCC axons (single asterisks) project posteriorly to join ipsilateral intersegmental nerves (ISNs) while RP2 axons (double asterisks) project anteriorly to join the ISNs. pCC axons are not indicated. **(B) **Comparison of RP2/aCC-enhanced (B1) and RP2-enhanced (B2) GFP labeling patterns (see Results) at 8 h-APF. The RP2/aCC-enhanced pattern was used to track neurons through metamorphosis; the RP2-enhanced pattern was used for cell culture experiments. Scale bars: 15 μm.

We used the Gal4/UAS system combined with FLP/FRT-mediated excision (see Materials and methods) to maintain postembryonic expression of membrane-bound GFP in RP2 and aCC (anterior corner cell) motoneurons and, less strongly (see below), pCC (posterior corner cell) interneurons. These neurons occur in segmentally repeated, bilateral pairs along the midline of the ventral ganglion and are readily identifiable *in situ *by their characteristic locations and projections [59-61] (Figure 2A). Their somata lie near the dorsal surface of the ventral ganglion, with aCCs and pCCs located immediately anterior to the RP2s of the next posterior neuromere. The motoneurons' axons exit the central nervous system via the ipsilateral intersegmental nerve (Figure 2A). In third instar larvae and young prepupae, GFP-labeled RP2s, aCCs and pCCs were present in both thoracic and abdominal neuromeres (data not shown) but, for simplicity, we restricted the present study to abdominal neuromeres A1 to A7.

The reporter lines used for this study produced two distinct patterns of neuronal labeling in third instar larvae and young prepupae, prior to the onset of metamorphic changes; conveniently, one pattern was advantageous for *in vivo *experiments while the other was advantageous for cell culture experiments. All animals were reared at 25°C unless indicated otherwise. In the first pattern ('RP2/aCC-enhanced'), GFP labeling was strong in both RP2s and aCCs, with all or nearly all of these motoneurons labeled throughout the abdominal central nervous system (for example, Figures 2A, B1 and 3A; Table 1). There was also variable and weaker labeling of a small proportion of pCC interneurons and some unidentified neurons (for example, Figures 2A, B1 and 3A3). The RP2/aCC-enhanced labeling pattern was observed in three conditions: all animals with two copies of *UAS-FLP*, *Act5C>y+>Gal4 *on chromosome 2 or 3; all animals with one copy of *UAS-FLP*, *Act5C>y+>Gal4 *on chromosome 2 or 3, reared at 29°C until the third instar; or in approximately half [62] of animals in which *UAS-FLP *was on chromosome 1 (see below). Animals with the RP2/aCC-enhanced phenotype from the first two categories were used to track neuronal fates during metamorphosis.

**Figure 3:**
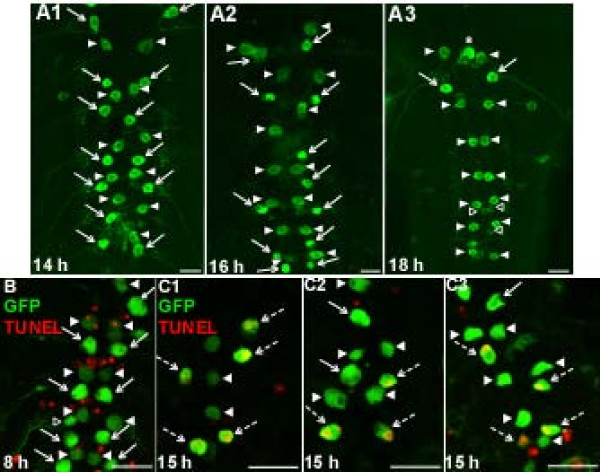
**RP2 disappearance occurs by programmed cell death**. **(A) **Images showing ventral ganglia removed from animals at 14, 16 and 18 h-APF. At 14 h-APF, nearly the full complement (2 per neuromere; Table 1) of abdominal RP2s and aCCs were identifiable by location and GFP labeling. By 16 h-APF, many RP2s were missing or were rounded and condensed. By 18 h-APF, only the RP2s in neuromere A1 remained. In contrast, aCCs persisted throughout this period. Asterisk in (A3) marks an example of an unidentified GFP-labeled neuron. **(B, C) **GFP labeling (green) and TUNEL labeling (indicating DNA fragmentation; red) in mid-abdominal neuromeres. (B) At 8 h-APF, many TUNEL-positive nuclei were present but no RP2s or aCCs were TUNEL-positive. (C1-C3) Images from three different animals at 15 h-APF. TUNEL-positive RP2s are marked by dashed arrows. Scale bars: 20 μm.

**Table 1:** Number of GFP-labeled motoneurons (mean ± standard deviation) in abdominal neuromeres A1 and A2 to A7 from 12 to 20 h-APF.

	Neuromere A1		Neuromeres A2 to A7	
		
	aCC	RP2	aCC	RP2
12 h-APF (14)	1.9 ± 0.3	1.9 ± 0.3	11.6 ± 0.6	11.8 ± 0.6
14 h-APF (20)	2.0 ± 0.0	1.9 ± 0.3	10.9 ± 1.1	10.7 ± 1.6
16 h-APF (21)	1.9 ± 0.2	1.8 ± 0.4	10.4 ± 1.0	4.6 ± 3.1^a^
18 h-APF (16)	1.9 ± 0.3	1.9 ± 0.3	10.6 ± 1.1	2.2 ± 2.5^a^
20 h-APF (10)	2.0 ± 0.0	2.0 ± 0.0	10.6 ± 1.0	0.1 ± 0.3^a^

The maximum possible total number of motoneurons was two aCCs and two RP2s in A1, and 12 aCCs and 12 RP2s in A2 through A7. Number of animals (n) is given in parentheses. ^a^Significant difference between number of aCC and RP2 motoneurons in A2 through A7, at the times shown (*P *< 0.0001; Student's paired *t*-test, two-tailed); the number of RP2s was first significantly reduced at 16 h-APF. The number of aCC and RP2 motoneurons in A1 was stable over this time period.

In the second labeling pattern ('RP2-enhanced'), RP2s were strongly GFP-labeled in third instar larvae and young prepupae, but the frequency and intensity of labeling of aCCs, pCCs and unidentified neurons were reduced compared to the RP2/aCC-enhanced pattern. The RP2-enhanced labeling pattern occurred in approximately half the animals with *UAS-FLP *on chromosome 1 (see above) [62]. Figure 2B compares the two GFP labeling patterns observed in animals of this genotype; we did not investigate potential causes of the two labeling patterns. Animals with the RP2-enhanced GFP labeling pattern were selectively used for cell culture experiments investigating RP2's response to 20E (see below).

In both labeling patterns, GFP labeling was sometimes absent from individual neurons, including RP2s (for example, Figures 2B2 and 3A; Table 1), perhaps due to variable efficacy of the Gal4/UAS and FLP/FRT method [58,63].

### Loss of RP2s during metamorphosis occurs by programmed cell death

Previous reports documented an intense period of PCD in the ventral ganglion during the first 24 h-APF [2,17]. To investigate RP2 and aCC fates during this period, we first compared ventral ganglia at 8 and 30 h-APF; in this and all subsequent *in vivo *experiments, only animals with the RP2/aCC-enhanced GFP labeling pattern were used. At 8 h-APF, the repeating segmental pattern of RP2s and aCCs was observed in 100% of ventral ganglia (n = 23 animals; for example, Figures 2 and 3B); in contrast, in all ventral ganglia examined at 30 h-APF, aCCs were present while RP2s were missing, except in neuromere A1 (n = 18 animals) [62]. To investigate this phenomenon more precisely, we quantified the number of RP2s and aCCs in neuromeres A1 to A7 at 2-hour intervals from 12 to 20 h-APF (Table 1). In A1, the maximum possible number of GFP-labeled RP2s and aCCs was two each; however, because GFP labeling was occasionally absent (see above), the mean number of RP2s and aCCs in A1 was slightly less than two. These values did not change between 12 and 20 h-APF (Table 1), confirming that RP2s and aCCs persisted in A1 throughout this period. A different pattern was observed in neuromeres A2 to A7, in which the maximum possible total number of GFP-labeled RP2s and aCCs was 12 each. Between 12 to 20 h-APF, the number of aCCs remained stable while the number of RP2s declined precipitously, with the largest loss occurring between 14 and 16 h-APF (Table 1). Representative images of these patterns are shown in Figure 3A. During the peak period of RP2 loss, many RP2s appeared rounded and condensed (for example, Figure 3A2), consistent with the possibility that these neurons were dying.

To test the hypothesis that the loss of RP2s between 14 and 20 h-APF resulted from PCD rather than, for example, loss of GFP expression, we used the TUNEL method (terminal deoxynucleotidyl transferase mediated dUTP nick end labeling; see Materials and methods) to label fragmented DNA, a hallmark of PCD [64]. Ventral ganglia examined between 6 and 10 h-APF exhibited many TUNEL-positive nuclei, but no TUNEL-positive RP2s (for example, Figure 3B; n = 6 animals). However, at 15 h-APF, every ventral ganglion examined had one or more TUNEL-positive RP2s (Figure 3C; n = 10 animals). At no time during the observation period did we see TUNEL-positive aCCs (n = 16 animals). We conclude from these data that RP2s in neuromeres A2 to A7 underwent PCD between approximately 14 and 20 h-APF. In contrast, the RP2s in A1, and all abdominal aCCs, survived, thereby serving as convenient positive controls in subsequent experiments.

### Genes and proteins involved in RP2 death

**Figure 4:**
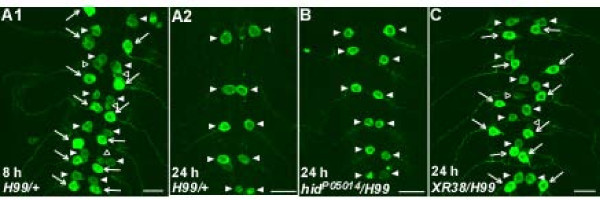
**Reaper is necessary for RP2 death**. **(A) ***H99/+ *animals (heterozygous for a deletion that removes *reaper, hid*, and *grim*) showed normal patterns of GFP-labeled neurons at 8 h-APF (A1) and RP2 death and aCC survival at 24 h-APF (A2). **(B) **A *hid^P05014^/H99 *animal (*hid *loss of function mutation) showed normal RP2 death and aCC survival at 24 h-APF. **(C) **In a *XR38/H99 *animal (*reaper *deletion), RP2s failed to undergo PCD and were present along with aCCs at 24 h-APF. All images are from mid-abdominal neuromeres. Scale bars: 20 μm.

To further characterize the death of RP2s during metamorphosis, we tested for the involvement of a subset of the many genes and proteins implicated in PCD in *Drosophila *[31]. The PCD of cells, including neurons[26,65], depends on proteins encoded by the death-activating genes *reaper, hid*, and *grim *[49-51]. We therefore tested whether these genes were involved in the death of RP2s. In an *H99/+ *mutant background in which *reaper, hid*, and *grim *were reduced to one copy [51], RP2s in neuromeres A2 to A7 died normally; they were present at 8 h-APF (Figure 4A1; n = 5 animals) but absent by 24 h-APF, while all aCCs survived (Figure 4A2; n = 17 animals). Likewise, in a *hid^P05014^/H99 *background that removes *hid *function and reduces *reaper *and *grim *to one copy[50,51,51], RP2s in A2 to A7 died while the aCCs survived normally (Figure 4B; n = 12 animals). In contrast, in *Df(3L)XR38/H99 *animals, in which *reaper *was eliminated and *hid *and *grim *were reduced to one copy [65], RP2s failed to undergo PCD and were present along with aCCs at 24 h-APF (Figure 4C; n = 9 animals). In the above experiments, RP2s in neuromere A1 behaved like aCCs (data not shown). These results suggested an exclusive requirement for *reaper *in the PCD of RP2s in neuromeres A2 to A7.

**Figure 5:**
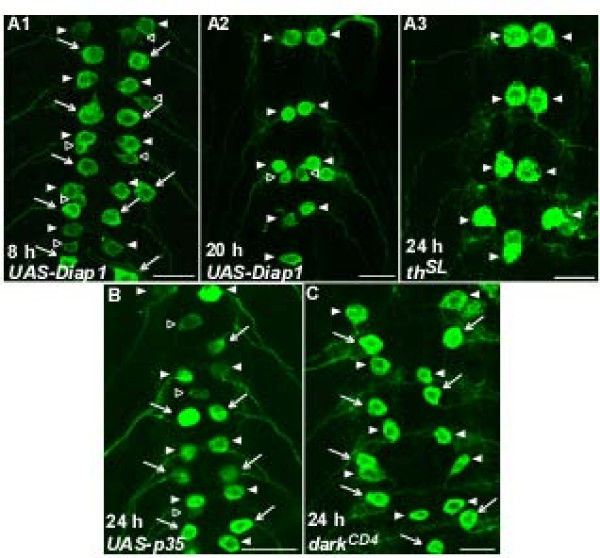
**Effects of perturbing Diap1, p35 and Dark on RP2 death**. **(A) ***UAS-Diap1 *animals showed normal patterns of GFP-labeled neurons at 8 h-APF (A1) and RP2 death and aCC survival at 20 h-APF (A2). (A3) RP2 death was likewise unimpaired in *th^SL ^*mutants, in which Diap1 inactivation by Reaper is suppressed. **(B) **Expression of p35 via *UAS-p35 *blocked the death of RP2s, which were still present at 24 h-APF. **(C) **In a *dark^CD4^/dark^CD4 ^*animal (homozygous for a *dark *hypomorphic allele), RP2s were still present at 24 h-APF. All images are from mid-abdominal neuromeres. Scale bars: 20 μm.

In most instances of developmental PCD, Reaper, Grim, and/or Hid normally antagonize Diap1 to liberate the apical caspase Dronc, which in turn activates effector caspases such as Drice [31,46,66,67]. Given the requirement of Reaper for RP2's death, we tested whether ectopic expression of Diap1 in RP2s would block PCD - for example, as it does in larval *Drosophila *salivary glands [67]. In *UAS-Diap1 *animals, all RP2s were present at 8 h-APF, as expected (Figure 5A1; n = 5 animals). At 20 h-APF, RP2s in A2 to A7 were missing while aCCs survived (Figure 5A2; n = 15 animals), suggesting that Diap1 expression could not antagonize the death of RP2s. As a positive control, we replicated the finding that ectopic expression of Diap1 in larval salivary glands prevented degeneration (data not shown) [62]. We further investigated the involvement of Diap1 in animals homozygous for a dominant *diap1 *mutation, *thread^SL ^*(*th^SL^*). The *th^SL ^*allele produces a Diap1 protein with an amino acid substitution in the BIR-1 domain, which suppresses Diap1 inactivation by Reaper [68]. However, RP2 death proceeded normally in *th^SL ^*homozygotes (Figure 5A3; n = 8 animals), just as in *UAS-Diap1 *animals. In all cases, RP2s in neuromere A1 behaved like aCCs (data not shown). These results, using two independent genetic manipulations, suggested that Diap1 may not play a role in the death of RP2s in A2 to A7; a similar result was reported for the Reaper-dependent PCD of vCrz neurons during *Drosophila *metamorphosis [17].

To test the roles of caspases in RP2 death, we mis-expressed the viral caspase inhibitor p35 using a *UAS-p35 *responder. p35 blocks PCD by acting as a cleavage substrate for the effector caspase Drice [69,70]. In the *UAS-p35 *genetic background, RP2s were present at 8 h-APF, as expected (n = 11 animals; data not shown). At 24 h-APF, RP2s were still present in all *UAS-p35 *animals examined (Figure 5B; n = 17 animals), consistent with a requirement for effector caspase activity for the PCD of RP2s.

Activation of the apical caspase Dronc requires assembly of the *Drosophila *Apaf-1 homologue, Dark [71], into apoptosomes, with *dark *mutations phenocopying null mutations in *dronc *[41] We tested the requirement for *dark *in RP2 death using animals homozygous for the hypomorphic *dark^CD4 ^*allele. At 24 h-APF, RP2s were still present in *dark^CD4 ^*mutants (Figure 5C; n = 8 animals), suggesting that apoptosome assembly involving Dark and Dronc is required for the PCD of RP2s.

In summary, of the subset of *Drosophila *PCD-related genes and proteins that we tested, several were implicated in the death of RP2s in neuromeres A2 to A7 during metamorphosis. The only exception was Diap1 (see Discussion).

### RP2 death is triggered directly and cell-autonomously by 20E

The time course of the death of RP2s in neuromeres A2 to A7 (Table 1) closely matches that of the degeneration of larval salivary glands, which was shown in tissue culture to be triggered directly by the rise of ecdysteroids during the prepupal pulse [66]. In *Manduca*, the segment-specific PCD of APR motoneurons at pupation is likewise triggered directly and cell-autonomously by the rise in ecdysteroids during the prepupal pulse, as demonstrated by placing fluorescently labeled APRs in primary cell culture and exposing them to 20E [56]. We therefore tested the hypothesis that prepupal ecdysteroids act directly on *Drosophila *RP2s to trigger PCD.

**Figure 6:**
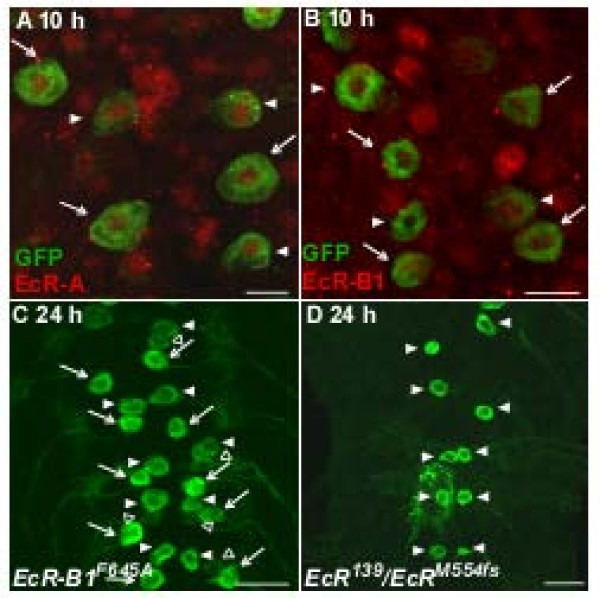
**RP2 death requires ecdysteroid receptor function**. **(A, B) **GFP labeling (green) and EcR immunoreactivity (red). At 10 h-APF, just prior to head eversion, both RP2s and aCCs exhibited low levels of nuclear EcR-A (A) and EcR-B1 (B). **(C) **In *UAS-EcR-B1^F645A ^*(dominant-negative EcR-B1 mutation expressed in the neurons), RP2s were still present at 24 h-APF. **(D) ***EcR^139^/EcR^M554fs ^*(EcR-A null mutation) animals showed normal RP2 death and aCC survival at 24 h-APF. Scale bars: 10 μm in (A, B); 20 μm in (C, D).

If ecdysteroids act directly on RP2s to activate PCD, then RP2s should express ecdysteroid receptors at the appropriate time. Immunolabeling showed that both EcR-A and EcR-B1 were present in the nuclei of RP2s and aCCs in all abdominal segments at 8 to 10 h-APF, during the initial upswing of the prepupal pulse (Figure 6A, B; n = 6 and 5 animals, respectively). EcR labeling was qualitatively similar in all RP2s and aCCs, although only the RP2s in neuromeres A2 to A7 were fated to die. Levels of EcR-A and EcR-B1 in RP2s appeared low relative to their levels in co-processed ventral ganglia from late pupae or white prepupae (data not shown) [27,72]. Control experiments with primary antibody omitted eliminated labeling (data not shown). Antibodies specific to EcR-B2 are unavailable so this isoform was not examined.

We next tested the requirement for EcRs in RP2's death by misexpressing a dominant-negative EcR-B1 mutation that binds ecdysteroids but lacks transcriptional activation (*UAS-EcR-B1^F645A^*) [8,73]. This mutation competitively displaces all three endogenous EcR isoforms so cannot distinguish the roles of individual isoforms. In *UAS-EcR-B1^F645A ^*animals, RP2s failed to undergo PCD and were present along with aCCs when examined at 24 h-APF (Figure 6C; n = 9 animals), suggesting that EcR activation was required for PCD. To test the role of EcR-A, we examined the fate of RP2s in an *EcR-A *null mutant (*EcR^139^/EcR^M554Fs^*) [74,75]. RP2s were present in all ventral ganglia at 8 h-APF (data not shown; n = 3 animals) and missing in neuromeres A2 to A7 when examined at 24 h-APF, while aCCs and the RP2s in A1 survived (Figure 6D; n = 10 animals). This result suggests that RP2 death in A2 to A7 does not require EcR-A function, and that ecdysteroids instead act via one or both EcR-B isoforms.

Having found that EcRs were present in RP2s at 8 to10 h-APF, we tested whether PCD was a direct response to prepupal ecdysteroids. Cell cultures were prepared from abdominal portions of ventral ganglia at 8 h-APF, immediately prior to the prepupal pulse (Figure 1). In these experiments we utilized only animals with the RP2-enhanced GFP labeling pattern, in which the RP2s express GFP strongly and the frequency and intensity of GFP labeling in other neurons is reduced (see above). Pools of dissociated neurons were divided into paired 'sister' cultures; one culture received medium containing 6 μg/ml 20E while the other culture received medium without 20E.

Immediately after flooding the dishes (designated day 0) we photographed fields of neurons that contained one or more putative RP2s, as identified by characteristic soma size and strong GFP labeling. We use the term 'putative RP2s' because, although cultures were prepared from animals in which GFP expression was predominantly limited to RP2s, some proportion of GFP-labeled neurons in the cultures had other identities (for example, aCCs). For each putative RP2 included in the study, we also selected a non-GFP-labeled neuron in the same field of cells, of similar size and appearance as the putative RP2, as a control neuron (not shown). The same putative RP2s and control neurons were photographed at the end of the culture period (48 or 72 h). From photomicrographs, neurons were scored as alive or dead by morphological criteria (see Materials and methods) [24,56].

**Figure 7:**
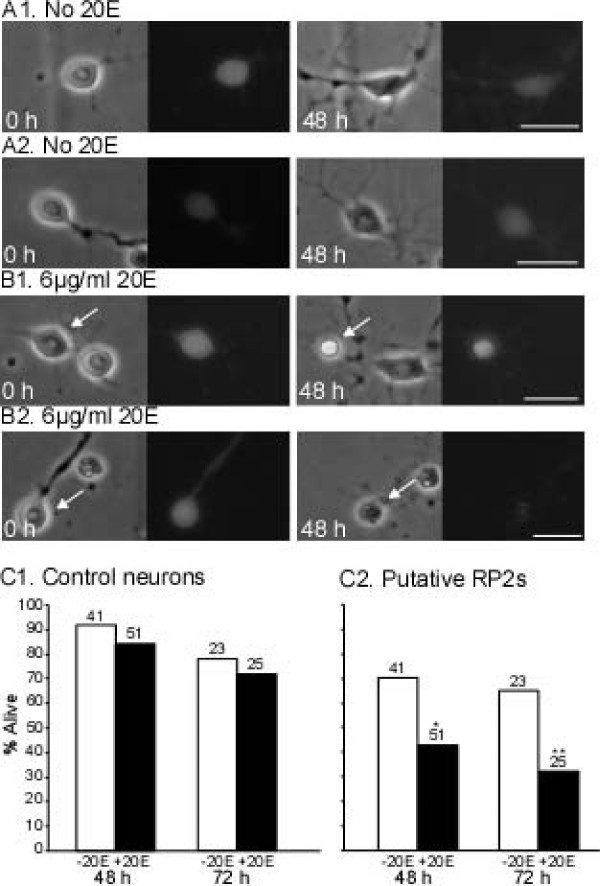
**20E-induced programmed cell death of putative RP2s in culture**. Sister cultures prepared at 8 h-APF were treated with medium with 6 μg/ml 20E (+20E) or no hormone (-20E). On day 0, putative RP2 neurons were identified by GFP expression and a similar-appearing neuron in the same optical field was selected to serve as a control for each putative RP2. At 48 or 72 h (independent experiments with four and two pairs of sister cultures, respectively) neurons were scored as dead or alive by morphological criteria. **(A, B) **Photomicrographs show putative RP2s at 0 h (left panels) and 48 h (right panels) in culture, under phase contrast (left) and GFP epifluorescence (right) optics. When multiple neurons were present, arrows mark putative RP2s. At 0 h, all putative RP2s had smooth, ovoid somata with well-defined nuclei. (A1, A2). In the absence of 20E, putative RP2s were still alive at 48 h, with GFP labeling and neurite outgrowth (B1, B2). In the presence of 20E, putative RP2s were dead by 48 h, as determined by morphological criteria; the neuron in (B1) was shrunken and phase-bright but retained GFP fluorescence, while that in (B2) was shrunken, had a fragmented outline and faint GFP fluorescence, and the nucleus was reduced to a small spot. **(C) **Histograms show percentage of neurons alive at the time indicated; n given above each bar. (C1) The percentage of control neurons alive at 48 or 72 h did not differ significantly between +20 and -20E cultures. (C2) At both 48 and 72 h, significantly fewer putative RP2s survived in cultures containing 20E (*P < 0.05, **P < 0.01, two-tailed Chi square test with Yates continuity correction). Scale bars: 10 μm.

The left panels of Figure 7A, B show four putative RP2s on day 0, with characteristic smooth, ovoid somata and well-defined nuclei. By definition, all neurons in the study were alive on day 0. The putative RP2s cultured without 20E were still alive 48 h later (Figure 7A). As illustrated in Figure 7A, neurons that were alive after 48 or 72 h typically maintained strong GFP labeling. Neurons were scored as dead if the soma was rounded and shrunken or showed significant degradation, and the nucleus was no longer discernable; many shrunken neurons were also phase bright. These morphologies can be seen in the putative RP2s in Figure 7B (right panels), which were cultured in 20E for 48 h. Among neurons scored as dead, GFP fluorescence was sometimes strong (Figure 7B1) and sometimes weak or absent (Figure 7B2). Accordingly, GFP fluorescence was not a useful criterion for neuronal viability and was used only to identify putative RP2s on day 0. Note that the rounded, condensed and strongly fluorescent appearance of some neurons dying in culture (Figure 7B1, right) resembled that of RP2s dying *in vivo *(Figure 3A2).

Figure 7C1 shows the percentage of control neurons alive after 48 or 72 h in culture, in the presence or absence of 20E. The hormone had no significant effect on the survival of control neurons at either time point; fewer neurons were alive in older cultures but this was seen with or without 20E. In contrast, 20E significantly decreased the survival of putative RP2s at both 48 and 72 h, with approximately twice as many putative RP2s dying in cultures containing 20E (Figure 7C2). Thus, the PCD-inducing effect of 20E was specific to putative RP2s and not a general effect on all cultured neurons. In other experiments in which putative RP2s were cultured from 'clear-gut' wandering larvae, 20E did not induce PCD of putative RP2s (data not shown). This is consistent with the developmental regulation of 20E's ability to trigger neuronal death, as also observed in *Manduca *[20].

### Propidium iodide confirmation of programmed cell death

**Figure 8:**
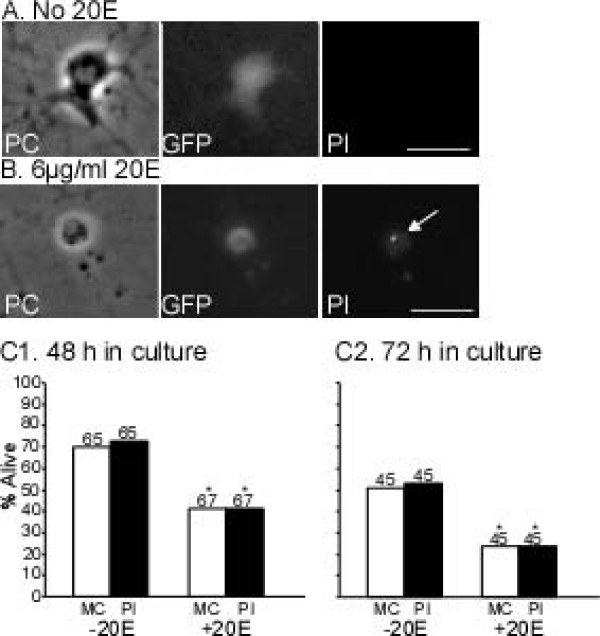
**Validation of morphological criteria for programmed cell death by propidium iodide staining**. Putative RP2s were cultured at 8 h-APF (independent experiments with six and four pairs of sister cultures, respectively) in medium with 6 μg/ml 20E (+20E) or no hormone (-20E). After 48 or 72 h, RP2s were independently scored as dead or alive using morphological criteria (MC) or PI staining. **(A, B) **Photomicrographs show putative RP2s under phase contrast (PC) and epifluorescence optics for GFP and PI (a marker for dead cells). (A) Putative RP2 cultured for 48 h without 20E. The neuron was alive by morphological criteria, exhibited GFP labeling and lacked PI staining. (B) Putative RP2 cultured for 48 h with 20E was dead by morphological criteria, exhibited GFP labeling and had strong nuclear PI staining (arrow). **(C) **Similar results were obtained by scoring putative RP2s by morphological criteria or PI staining after 48 h (C1) or 72 h (C2) in culture. Both methods showed that significantly fewer putative RP2s survived in cultures with 20E (*P < 0.01, two-tailed Chi square test with Yates continuity correction). Scale bars: 10 μm.

To independently assess the validity of the morphological criteria for PCD, we stained neurons with propidium iodide (PI) at the end of the culture period (48 or 72 h). Similarly to other vital stains such as acridine orange and trypan blue, PI is excluded from live neurons whereas membrane breakdown in dying or dead neurons allows PI to penetrate and fluorescently label nucleic acids[66,76], From photomicrographs, each putative RP2 was scored independently as dead or alive by morphological criteria and PI staining. All putative RP2s were alive on day 0 (data not shown). Figure 8A, B shows representative photomicrographs of putative RP2s after 48 h in culture without (Figure 8A) or with (Figure 8B) 20E. The putative RP2 in Figure 8A was alive by morphological criteria and lacked PI staining ('PI-negative') while the putative RP2 in Figure 8B was dead by morphological criteria and showed PI staining ('PI-positive'). Figure 8C compares the results from the two scoring methods. As in the previous experiments (Figure 7C2), 20E significantly reduced the percentage of putative RP2s alive after 48 h (Figure 8C1) or 72 h (Figure 8C2) in culture. The two scoring methods - morphological criteria and PI staining - yielded nearly identical results; neurons judged to be alive by morphological criteria were PI-negative whereas neurons judged to be dead by morphological criteria were PI-positive.

### Role of neuronal contacts in programmed cell death

**Figure 9:**
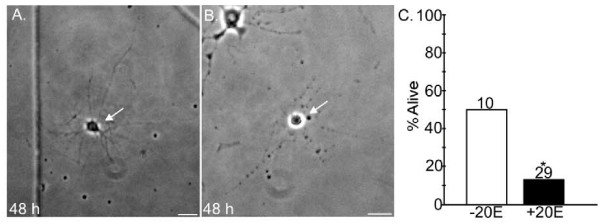
**Cell contact does not regulate putative RP2 fate in culture**. **(A, B) **Low-power photomicrographs show contact-free putative RP2s after 48 h in culture, in the absence (A) or presence (B) of 20E. The putative RP2 in (A) is alive and that in (B) is dead. Note absence of neuritic contact with other neurons. (C) Histogram shows percentage of putative RP2s alive at 48 or 72 h (data pooled from Figures 7 and 8); n given above each bar. Among these contact-free putative RP2s, significantly fewer survived in cultures containing 20E (*P = 0.028; Fisher's exact test). Scale bar: 10 μm.

In *Manduca*, contact with other neurons does not influence the ability of 20E to trigger segment-specific PCD of cultured motoneurons [56]. The current experiments utilized relatively high-density cultures, so many putative RP2s had somatic and/or neuritic contact with other neurons. To test the hypothesis that 20E acts directly on RP2s to trigger PCD, independently of neuronal contact, we analyzed separately a subset of putative RP2s that lacked contact with other neurons throughout the culture period (determined by high-magnification examination of photomicrographs). Figure 9A, B shows representative live and dead contact-free RP2s at 48 h, in the absence or presence of 20E, respectively. In both examples the neurons' somata and neurites (fragmented in Figure 9B) failed to contact adjacent neurons. The number of putative RP2s that lacked contacts was relatively small, so we pooled data from 48- and 72-h experiments (same data as Figures 7 and 8) to identify 39 contact-free putative RP2s. Figure 9C shows that, among these contact-free putative RP2s, significantly fewer survived when cultured with 20E, consistent with the idea that neuronal contact played no role in RP2's responses to 20E.

Taken together, the above results support the hypothesis that the rising phase of the prepupal pulse of ecdysteroids is the normal developmental trigger for PCD of RP2s in neuromeres A2 to A7 and that this effect is mediated directly.

## Discussion

Previous work in *Manduca *used a combination of *in vivo *and cell culture methods to establish that the segment-specific PCD of APR motoneurons during metamorphosis is triggered directly by ecdysteroids [20]. However, the relative lack of genetic tools available in *Manduca *motivated us to seek a comparable experimental system in *Drosophila*. After investigating a variety of potential reporters, we selected the *even-skipped *gene combined with other constructs (see Materials and methods) to drive GFP expression in two patterns that allowed us to track neuronal fates: RP2/aCC-enhanced and RP2-enhanced (Figure 2B). The lack of restriction of GFP expression to RP2s had advantages and disadvantages: one advantage is that the survival of aCCs under all conditions provided a powerful positive control for the death of RP2s in neuromeres A2 to A7. A disadvantage is that, even when using animals with the RP2-enhanced GFP labeling pattern, cell cultures contained some GFP-labeled neurons that were not RP2s. However, despite this complication, we readily detected 20E-induced PCD in culture (Figures 7 and 8). Further refinement of the reporter lines and genetic constructs could likely remedy these current limitations. The present study establishes RP2 motoneurons as tractable and informative subjects for investigating hormonally triggered PCD during *Drosophila *metamorphosis, in individually identified neurons of known function.

### A segmental subset of RP2s is eliminated by programmed cell death

During *Drosophila *metamorphosis, many larval motoneurons are remodeled or undergo PCD. We determined that the segmental subset of RP2s in neuromeres A2 to A7 dies during the larval-pupal transformation, with PCD beginning at approximately 14 to 16 h-APF, based on the disappearance of GFP-labeled RP2s in ventral ganglia (Table 1; Figure 3A). That the disappearance of GFP-labeled RP2s resulted from death rather than other causes (for example, cessation of GFP expression) was confirmed first by the presence of TUNEL labeling (indicating DNA fragmentation) in RP2s during the same time period (15 h-APF; Figure 3B) and, secondly, by the finding that genetic manipulations of known *Drosophila *PCD genes could block RP2's death (Figures 4 and 5; see below). Not all RP2s were TUNEL-positive at 15 h-APF, likely due to minor temporal variability in the PCD pathway within and between animals, also seen during motoneuron death in *Manduca *[77]. In all experimental conditions, aCC motoneurons in A1 to A7, and the RP2s in A1, survived this period of development (see Results).

### The involvement of programmed cell death genes and proteins in RP2s

It was beyond the scope of the present study to test the potential involvement of even a major fraction of genes implicated in *Drosophila *PCD. We instead focused on a small subset of representative genes. In *Drosophila*, the death-activating genes *reaper*, *hid*, and *grim *collectively or independently activate PCD, in many cases by antagonizing Diap1 [49-53]. Death of RP2s may depend exclusively on Reaper, as perturbation of *reaper *but not *hid *function impaired their deaths (Figure 4). We did not test the role of Grim directly but the finding that RP2 death was impaired in *reaper *mutants, despite one intact copy of *grim*, suggests that Grim is unnecessary. This is supported by studies suggesting that PCD in the developing central nervous system is normal in *grim *mutants [78].

In contrast to salivary glands[62,67], we were unable to block the death of RP2s by ectopic expression of Diap1 (Figure 5A2). Likewise, in *th^SL ^*homozygotes in which Reaper cannot inactivate Diap1, RP2 death was normal (Figure 5A3). Thus, Reaper-mediated PCD may be Diap1-independent in RP2s as well as vCrz neurons [17]. Reaper is multi-functional, including a role in mitochondrial permeabilization[79,80], which has been shown to be required for some developmental PCD [81]. Regardless of the involvement of Diap1, caspase activation appears necessary for RP2 death in A2 to A7, as shown by the ability of the viral caspase inhibitor p35 to preserve these neurons (Figure 5C). Also consistent with the role of caspases, RP2 death was blocked in animals with mutations in *dark *(Figure 5D), whose gene product is a key component of the apoptosome, where caspases are activated.

These results are consistent with a general requirement for H99 proteins, caspases and apoptosome function for embryonic and postembryonic PCD in the nervous system and other tissues of *Drosophila*[41,51], although cell-specific variation is observed [82]. For instance, both *reaper *and *hid *participate in death of larval salivary glands while only *reaper *is required for PCD of vCrz neurons [17,53]. Likewise, as discussed above, the role of Diap1 may vary in different situations.

### 20E activates the programmed cell death pathway in RP2s

Several independent lines of evidence suggest that the rising phase of the prepupal pulse of ecdysteroids triggers the death of RP2s in A2 to A7: the RP2s have nuclear EcRs at this time (Figure 6A, B), genetic disruption of EcR function by overexpression of a dominant-negative receptor blocks RP2 death (Figure 6C) and, in cell culture, 20E treatment delivered at the time of the prepupal pulse promotes selectively the death of putative RP2s (Figures 7 and 8). The requirement for EcR function is consistent with the overall role of ecdysteroids in driving metamorphosis [7], with different isoforms often associated with different developmental events. The EcR-A isoform mediates the PCD of type II neurons at the end of metamorphosis when ecdysteroids decline [27], whereas EcR-A is not required for RP2 death (Figure 6D). One or both EcR-B isoforms must therefore be responsible, but distinguishing between them was beyond the scope of this study. In *Drosophila*, EcR-B isoforms are implicated in many cases of neuronal remodeling [13-15] and neuronal death, including that of vCrz neurons in response to the late larval pulse of ecdysteroids [17]. It is notable that both doomed RP2s and persisting aCCs had qualitatively similar patterns of EcR-B1 expression (Figure 6A, B), so the simple presence or absence of receptor cannot account for their different fates. Molecular players other than EcR, including EcR's heterodimerization partner, ultraspiracle (USP)[83,84], and nuclear hormone co-regulators [85], are likely important for regulating neuron- and segment-specific actions of ecdysteroids.

The prepupal pulse of ecdysteroids begins at about 10 h-APF (Figure 1) and the death of RP2s begins between 14 and 16 h-APF (Table 1; Figure 3). This delay is consistent with typical latencies for EcR-induced cellular changes mediated via gene transcription [86]. We tested the hypothesis that the rise in the prepupal pulse of ecdysteroids triggers RP2 death by preparing neuronal cultures at 8-h APF and maintaining them with or without 20E for 48 or 72 h (Figures 7 and 8). In the absence of a reporter line that restricted GFP expression to RP2s, we used animals with the RP2-enhanced GFP labeling pattern in which most but not all GFP-labeled neurons were RP2s. Furthermore, due to variation in the exact site of the cut made to separate the thoracic and abdominal regions of the ventral ganglion before dissociating neurons, some RP2s from neuromere A1 may have been included in some cultures. Both of these factors could potentially impair the ability to detect a 20E effect. However, the ability of 20E to induce PCD of putative APRs was robust in each of the four independent experiments shown in Figures 7C2 and 8C2; in all cases, significantly more putative RP2s died in the presence than in the absence of 20E. Furthermore, this effect was specific to these neurons as 20E had no significant effect on the survival of control neurons in the same culture dishes. Results were essentially identical when using morphological criteria or PI staining to score neurons as alive or dead (Figure 8). Importantly, given that 20E exposure was maintained for the entire culture period, the rise in ecdysteroids during the prepupal pulse appears sufficient to trigger RP2 death, without requiring a subsequent decline in ecdysteroids. The same result was obtained for cultured *Manduca *motoneurons [56].

Some experimental limitations should be noted. Even after a 72-h exposure to 20E, some putative RP2s failed to degenerate (Figures 7 and 8); because the percentage of dying neurons increased between 48 and 72 hours, a longer culture period may have allowed more neurons to die. Conversely, some putative RP2s died when cultured in the absence of 20E; although all cultures were prepared at 8 h-APF, it is possible that some RP2s were already committed to PCD due to asynchrony when the prepupal pulse began. The identical phenomena were observed in *Manduca*, even with unambiguous identification of specific motoneurons by retrograde fluorescent labeling from their target muscle[24,54,56].

The present study identified the hormonal trigger and a few of the intermediate steps required for RP2's death during metamorphosis, but further experiments are required to determine to what extent the specific pathway for ecdysteroid-activated PCD in RP2s matches that in other *Drosophila *cells and tissues. Ecdysteroids and ecdysteroid-regulated early genes up-regulate Reaper and Dronc in larval midgut and salivary glands [87-91] and transcriptional co-activators or repressors that operate outside of EcR signaling (for example, Forkhead and Med24) may also be critical for the timing and execution of PCD [92,93]. One intriguing but unanswered question is how segmentally iterated neurons such as RP2 exhibit segment-specific responses to a generalized hormonal cue.

## Conclusions

This study introduces RP2 motoneurons as a useful model for investigating how ecdysteroids regulate postembryonic PCD in *Drosophila *in neurons of known function. Data from *in vivo *and cell culture experiments suggest that the rise in ecdysteroids during the prepupal pulse acts directly, via EcR-B isoforms, to activate PCD in RP2s in abdominal neuromeres A2 to A7, while sparing RP2s in A1. Genetic manipulations suggest that RP2's death requires Reaper function, apoptosome assembly and Diap1-independent caspase activation. RP2s offer a valuable 'single cell' approach to the molecular understanding of neuronal death during insect metamorphosis and, potentially, of neurodegeneration in other contexts [94].

## Materials and methods

### ***Drosophila *****rearing**

*Drosophila *were reared in 25 × 95 mm polystyrene vials on standard cornmeal-yeast-agar medium [95] with propionic acid (4.71 μl/ml; Sigma-Aldritch, St Louis, MO, USA) and tegosept (7 μg/ml; Genesee Scientific, San Diego, CA, USA), on a 12 h light/12 h dark photoperiod. The rearing temperature was 25°C unless noted otherwise (see below). Animals were collected at the onset of pupariation, held at 25°C on H_2_O-moistened tissues in culture dishes, and staged by h-APF.

### Stocks and crosses

All stocks used to maintain reporter gene expression in RP2s and aCCs were obtained from the *Drosophila *Stock Center (Bloomington, IN, USA). A GFP reporter was driven in RP2s and aCC (and pCCs; see Results) using flies homozygous for the *RN2-Gal4*, *UASmCD8GFP *transgenes recombinant (second or third chromosome). In these flies, Gal4, driven by upstream promoter fragments of the *even-skipped *gene, drove expression of membrane-bound GFP in RP2s and aCCs exclusively in embryos [60,96]. We crossed these to flies that were either homozygous for a *UAS-FLP*, *Act5C>y+>-Gal4 *transgene recombinant (second or third chromosome) or doubly homozygous for a *UAS-FLP *(first chromosome) and *Act5C>y+>Gal4, UAS-GFP *transgenes(s) (second chromosome). In this arrangement, Gal4 fell under the control of the *Actin5C *promoter following the removal of an FRT cassette by *UAS-*driven FLP-recombinase[58,63,97]. F1 progeny from these crosses maintained postembryonic GFP expression in RP2s and aCCs and were used for experiments.

For some experiments, including those using *reaper, hid, dark, diap1 *and *EcR *mutations, animals carrying both a *UAS-FLP*, *Act5C>y+>Gal4 *and *RN2-Gal4*, *UASmCD8GFP *recombinant chromosome within the appropriate mutant background were generated to maintain GFP expression in RP2s and aCCs. Expression was enhanced by rearing animals at 29°C from oviposition to the beginning of the third instar. The following deficiencies and alleles were used: *Df(3L)H99 *(*H99*) (*Drosophila *Stock Center), *hid^P05014^, thread^SL^*, and *Df(3L)XR38 *(*XR38*) (generously supplied by Dr Kristen White, Massachusetts General Hospital), *dark^CD4 ^*(*Drosophila *Stock Center) [43], *EcR^139 ^*and *EcR^M554Fs ^*(generously supplied by Dr Michael Bender, University of Georgia)[74,75,98]. The *XR38/H99 *transheterozygous combination produces deletion of *reaper *[68] while *hid^P05014^/H99 *causes a loss of *hid *function[50,51,65]. The *EcR^139^/EcR^M554Fs ^*transheterozygote results in an *EcR-A *null. For misexpression experiments, progeny containing at least one *UAS-FLP*, *Act5C>y+>Gal4 *and *RN2-Gal4*, *UASmCD8GFP *recombinant chromosome along with either *UAS-p35 *[70], *UAS-Diap1 *[99] or *UAS-EcR-B1^F645A ^*[73,100] were used.

### Immunolabeling and TUNEL processing

Using fine forceps, ventral ganglia were dissected under ice-cold PBS (pH 7.2) and fixed overnight in 4% paraformaldehyde (Electron Microscopy Sciences, Hatfield, PA, USA) in PBS at 4°C. Immunolabeling of GFP was performed using a rabbit anti-GFP polyclonal antibody (1:500) and an Alexa Fluor 488-conjugated goat anti-rabbit IgG secondary antibody (1:200; Invitrogen, Eugene, OR, USA). EcR immunolabeling was performed with monoclonal antibodies 15G1a (1:10; EcR-A) or AD4.4 (1:10; EcR-B1) [5] (Developmental Studies Hybridoma Bank developed under the auspices of the NICHD and maintained by the University of Iowa, Iowa City, IA, USA). Ventral ganglia were subjected to TUNEL using the Apop Tag ® Red In Situ Apoptosis Detection Kit (Chemicon Intl, Temecula, CA, USA). Ganglia were imaged on either a Bio-Rad Radiance 2100 (Bio-Rad, Hercules, CA, USA) or Zeiss Pascal LSM5 confocal microscope using a 488 nm excitation maximum argon laser (Alexa Fluor 488) or 543 nm excitation maximum helium-neon laser (rhodamine). Images were processed and converted into 8 bit bitmap images using ImageJ 1.37 (NIH, Bethesda, MD, USA).

### Cell culture

Animals were collected at 8 h-APF, immediately prior to the prepupal pulse (Figure 1). The dorsal surfaces of intact prepupae were examined under a Zeiss Axiovert 25 inverted microscope and those with strong GFP expression in RP2s, and weak or no GFP expression in aCCs (see Results), were selected for experiments. Neuronal cultures were prepared using methods modified from those described previously [12,101]. Prepupae were sterilized in 95% ethanol, rinsed in sterile H_2_O and dissected under a laminar flow hood in glass wells (pre-sterilized with 95% ethanol) containing modified *Drosophila *defined medium (DDM2) consisting of Ham's F-12 DMEM (high glucose; Irvine Scientific, Santa Ana, CA, USA) supplemented with 1.2 mg/ml sodium bicarbonate, 20 mM HEPES, 100 µM putrescine, 30 nM sodium selenite, 20 ng/ml progesterone, 50 µg/ml insulin, 100 µg/ml transferrin (Sigma-Aldrich) and 1% Pen-Strep (Invitrogen). Ventral ganglia were removed using fine forceps after which the abdominal portion was severed from the thoracic portion using a sterile 28 gauge needle (Monoject, St Louis, MO, USA). Due to the small size of the tissue, the exact location of the cut varied within the range of the third thoracic neuromere to the second abdominal neuromere.

Either six or eight abdominal ventral ganglia were pooled, divided evenly into two groups, enzymatically treated in Rinaldini's saline [102] containing 50 U/ml of papain and 1.32 mM L-cysteine (Sigma) for 15 minutes, rinsed three times in DDM2 and mechanically dissociated using a fine-tipped, fire-polished Pasteur pipette. This produced two 20-μl suspensions, each of which was dispensed into a well formed by punching an 8-mm hole into a 35-mm culture dish and attaching with Sylgard (Dow Corning Corp., Midland, MI, USA) an alphanumeric gridded coverslip (Bellco Biotechnology, Vineland, NJ, USA) coated with Concanavalin A (200 μg/ml) (Sigma) and laminin (3.55 μg/ml; Invitrogen). Each well contained 80 μl DDM2, resulting in a final volume of 100 μl. Pairs of cultures prepared in this manner were termed 'sister cultures.' After 30 minutes, each dish was flooded with a 3:1 mixture of DDM2 and unconditioned neurobasal medium (Invitrogen) supplemented with B27 (20 μl/ml; Invitrogen) to a final volume of 3 ml. One culture of each sister pair received medium containing 20E (Sigma) at a final concentration of 6 μg/ml while the control culture received 20E-free medium. Cultures were maintained in a humidified 23°C, 5% CO_2 _ambient-O_2 _incubator.

Cultured neurons were photographed after flooding (day 0) and again at 48 or 72 h *in vitro*. Fields containing one or more putative RP2 neurons, identified by their strong GFP expression (see Results), were selected randomly and photographed under phase contrast and GFP epifluorescence optics using a Nikon Coolpix 4500 digital camera attached to a Zeiss Axiovert 25 inverted microscope. Typically, 5 to 13 fields were photographed per dish. Using the gridded coverslips, the same fields were relocated and re-photographed 48 or 72 h later. For PI (Invitrogen) staining, PI was added to cultures at a final concentration of 20 μg/ml after 48 or 72 h *in vitro*. Live cells exclude PI from their somata while the nucleic acids of dead cells label strongly. PI-treated cultures were photographed as described above and also under rhodamine epifluorescence optics.

### Scoring neurons as alive or dead

Cultured neurons were scored as alive or dead by morphological criteria [24,56]. Live neurons had smooth somata, ovoid shapes and well-defined nuclei (see Results). Only putative RP2s that fulfilled these criteria were entered into the study on day 0. We also examined the effect of 20E on control neurons; for each putative RP2 selected on day 0, a GFP-negative neuron of similar size and appearance to the putative RP2 and located in the same photographic field was entered into the study. By definition, all putative RP2s and control neurons were alive on day 0. At the end of the culture period (48 or 72 h), an observer blind to treatment condition was provided digital images (phase-contrast and GFP fluorescence) of each putative RP2 or control neuron on day 0 and on the final day of culture. Neurons were scored as dead if the soma was rounded and shrunken or showed significant degradation, and the nucleus was no longer discernable; many dead neurons also became uniformly phase bright (see Results). For cultures stained with PI, the observer was also provided a digital image taken under rhodamine fluorescence. These neurons were scored as dead or alive by morphological criteria and independently by the presence or absence of PI staining.

Chi square with Yates continuity correction, or Fisher exact statistical tests were used to compare the proportions of neurons surviving under different conditions.

## Abbreviations

20E: 20-hydroxyecdysone; aCC: anterior corner cell; APR: accessory planta retractor; Dark: *Drosophila *Apaf-1 related killer; DDM2: *Drosophila *defined medium; Diap1: *Drosophila *inhibitor of apoptosis protein 1; Dronc: *Drosophila *Nedd2-like caspase; EcR: ecdysone receptor; GFP: green fluorescent protein; h-APF: hours after puparium formation; PBS: phosphate buffered saline; pCC: posterior corner cell; PCD: programmed cell death; PI: propidium iodide; *th: thread*; TUNEL: terminal deoxynucleotidyl transferase mediated dUTP nick end labeling; vCrz: ventral corazonin.

## Competing interests

The authors declare that they have no competing interests.

## Authors' contributions

AW participated in the design and interpretation of the study, performed all of the experiments and drafted the manuscript. JW participated in design and interpretation of the study and manuscript writing. Both authors read and approved the final manuscript.
